# Retraction: Synthesis, characterization, and theoretical studies of the photovoltaic properties of novel reactive azonitrobenzaldehyde derivatives

**DOI:** 10.1039/d5ra90041g

**Published:** 2025-04-04

**Authors:** Hitler Louis, Izubundu B. Onyebuenyi, Joseph O. Odey, Azuaga T. Igbalagh, MaryJane T. Mbonu, Ededet A. Eno, Anthony M. S. Pembere, Offiong E. Offiong

**Affiliations:** a Computational and Bio-Simulation Research Group, Department of Pure and Applied Chemistry, University of Calabar Calabar Nigeria louismuzong@gmail.com; b Department of Pure and Applied Chemistry, Faculty of Physical Sciences, University of Calabar Calabar Nigeria; c Department of Chemical Sciences, Federal University of Wukari Wukari Nigeria; d Department of Physical Sciences, Jaramogi Oginga Odinga University of Science and Technology Bondo Kenya

## Abstract

Retraction of ‘Synthesis, characterization, and theoretical studies of the photovoltaic properties of novel reactive azonitrobenzaldehyde derivatives’ by Hitler Louis *et al.*, *RSC Adv.*, 2021, **11**, 28433–28446, https://doi.org/10.1039/D1RA05075C.

We the authors hereby wholly retract this *RSC Advances* article due to concerns with the reliability of the data.

The experimental work reports simulated NMR spectra, and the mass spectra are unclear and not compatible with the molecules reported in the text. The HUMO orbital and LUMO orbital images in Fig. 7B are identical, as well as the electrostatic potential images for Fig. 7C and D.

The authors have not been able to provide the original mass spectrometry and NMR data.

In addition, a number of references were inappropriately replaced with self-citations by the authors during the revision process.

Given the significance of these concerns, the Editor has lost confidence that the findings presented in this paper are reliable.

The authors were informed; Joseph Omang Odey and Hitler Louis agreed with the retraction, and the others have not responded to any correspondence regarding the retraction.

Signed: Joseph Omang Odey and Hitler Louis (on behalf of other authors)

Date: 28th March 2025

Retraction endorsed by Laura Fisher, Executive Editor, *RSC Advances*

